# The effect of preserving pregnancy in cervical cancer diagnosed during pregnancy: a retrospective study

**DOI:** 10.1186/s12905-022-01885-w

**Published:** 2022-07-25

**Authors:** Zuoxi He, Chuan Xie, Xiaorong Qi, Zhengjun Hu, Yuedong He

**Affiliations:** 1 Department of Gynecology and Obstetrics, West China Second University Hospital, Sichuan University, Chengdu, Sichuan Province People’s Republic of China; 2 Key Laboratory of Birth Defects and Related Diseases of Women and Children, Ministry of Education, West China Second University Hospital, Sichuan University, No. 20 Section Three, South Renmin Road, Chengdu, 610041 Sichuan Province People’s Republic of China

**Keywords:** Cancer during pregnancy, Cervical cancer, Maternal and perinatal outcomes, Pregnancy-preserving

## Abstract

**Objective:**

Cervical cancer diagnosed during pregnancy is a rare event, and data regarding efficacy of cancer treatment during pregnancy is limited. This study aimed to assess the safety of continuation of the pregnancy for mother and fetus when concomitantly diagnosed with cervical cancer.

**Methods:**

This study retrospectively analyzed all cervical cancer patients diagnosed while pregnant or immediately postpartum, inclusive from Jan 2010 to June 2019 at our institute. Patient clinical details and follow-up were obtained from hospital records.

**Results:**

The study comprised 40 patients with clinical cancer stages of IA1 (1/40, 2.5%); IB1 (15/40, 37.5%); IB2 (10/40, 25%); IIA (12/40, 30%); and IIB (2/40, 5%). There were 38 patients diagnosed during pregnancy, and 2 diagnosed in the postpartum period. Of the 38 patients, 17 were diagnosed in the first trimester, 13 in the second trimester, and 8 in the third trimester. 10 of 38 patients (26.3%) continued their pregnancy after learning of their diagnosis; 7 (70%) in the third trimester and 3 (30%) in the second trimester. The mean time from diagnosis to surgery in the patients who continued their pregnancy was 52.7 days, which was statistically significantly greater than the termination of pregnancy group (52.7 vs. 16.3 days, *P* < 0.01). Notably, there was no survival difference between the 2 groups (100% vs. 90.91%, *P* = 0.54), and none of the pregnant women who ultimately died had delayed treatment due to pregnancy. Similarly, the surgical estimated blood loss and operative duration comparison in the 2 groups were not significantly different.

**Conclusions:**

In the present study, the gestational age of pregnancy at the time of initial diagnosis of cervical cancer was an important determinant in the disease management. Continuation of the pregnancy when diagnosed with cervical cancer may not affect the oncologic outcome of the mother nor increase either surgical or obstetric complications. Additionally, the use of neoadjuvant chemotherapy did not threaten the health of the fetus. These results may be useful in counseling patients facing the diagnosis of cervical cancer during pregnancy.

## Introduction

Worldwide, cervical cancer ranks fourth for both incidence and mortality in female cancer [1]. In recent years, the incidence of cervical cancer has trended to younger age patients. In China, the newly diagnosed cervical cancer  under the age of 45 is 29.7 thousand [2]. Although uncommon, the diagnosis of cervical cancer in a woman who is pregnant is frightening and important. The incidence of cervical cancer diagnosed during pregnancy ranges from 1.4 to 4.6 per 100,000 [3], but the incidence is increasing as a result of later age of marriage and consequent later childbearing of modern women [4–6].

In theory, hormonal variation and local immunosuppression may induce human papillomavirus (HPV) virus reactivation during pregnancy [7], which raises concern that pregnancy might indirectly accelerate cervical cancer. Moreover, the increased uterine blood circulation and cervical dilatation during labor could potentially enhance tumor cell spread and accelerate the progression of cervical cancer [8]. However, recent studies have demonstrated that pregnancy does not affect the prognosis of the mothers and the neonatal outcomes are good [9–13].

When cervical cancer is diagnosed during pregnancy, whether the pregnancy should be continued and how to manage the cervical cancer remain controversial. In these circumstances, crucial questions will inevitably arise for both physician and pregnant patient: will the delayed operation be safe for mother and fetus; will the postponement of the operation accelerate the progress of tumor; or will neoadjuvant chemotherapy (NACT) harm the fetus? Because data regarding maternal and fetal prognosis in the management of cervical cancer diagnosed during pregnancy are limited, we retrospectively reviewed such cases diagnosed at West China Secondary University Hospital.

## Materials and methods

The study protocol was reviewed and approved by the ethics committee and the data inspectorate of West China Second University Hospital of Sichuan University. Ethical approval and patient consent were acquired and recorded in the patient medical record with witness signature. All ethical approval and consent procedures were approved by the Medical Ethical Committee of West China Second University Hospital, Sichuan University. Informed consent was obtained from all participants.

All cases of cervical cancer diagnosed during pregnancy from Jan 2010 through June 2019 were retrieved from the Anatomical Pathology Department at West China Second University Hospital, Sichuan University. All diagnoses were confirmed by pathological examination of the cervical biopsy. Nearly half of cervical cancers associated with pregnancy are diagnosed within 6 months following delivery, and women diagnosed in the postpartum period have worse survival than those diagnosed during pregnancy [14–16]. Therefore, strong consideration should be given to include investigation not only of patients diagnosed with cervical cancer during pregnancy, but also patients diagnosed within six months following delivery. Accordingly, our study included patients diagnosed during pregnancy and within six months following delivery. Cervical cancer was staged according to the standard of the International Federation of Gynecology and Obstetrics (FIGO) in 2009 [17]. At least two experienced gynecologic oncologists were involved in determining the clinical stage.

Patient clinical details and follow-up were obtained from hospital records, including sociodemographic, oncologic and obstetrical outcome data. Oncologic data included the clinical tumor stage, histology, HPV status, pathological features, therapeutic approach, operative duration, estimated blood loss (EBL), surgical complications and survival. Obstetrical outcome data included gestational age (GA) at initial diagnosis, GA at termination, delivery mode, fetal Apgar scores, newborn complications caused by antineoplastic therapy, and current status of neonates. All patients were followed by telephone.

Data were analyzed using software SPSS 25.0, the continuous variable was the mean ± standard deviation, and the independent sample mean was compared by T-test. The classified variables were analyzed by X^2^ test or rank sum test. *P* value < 0.05 was considered statistically significant.

## Results

### Patient sociodemographic characteristics at diagnosis

**Fig. 1:**
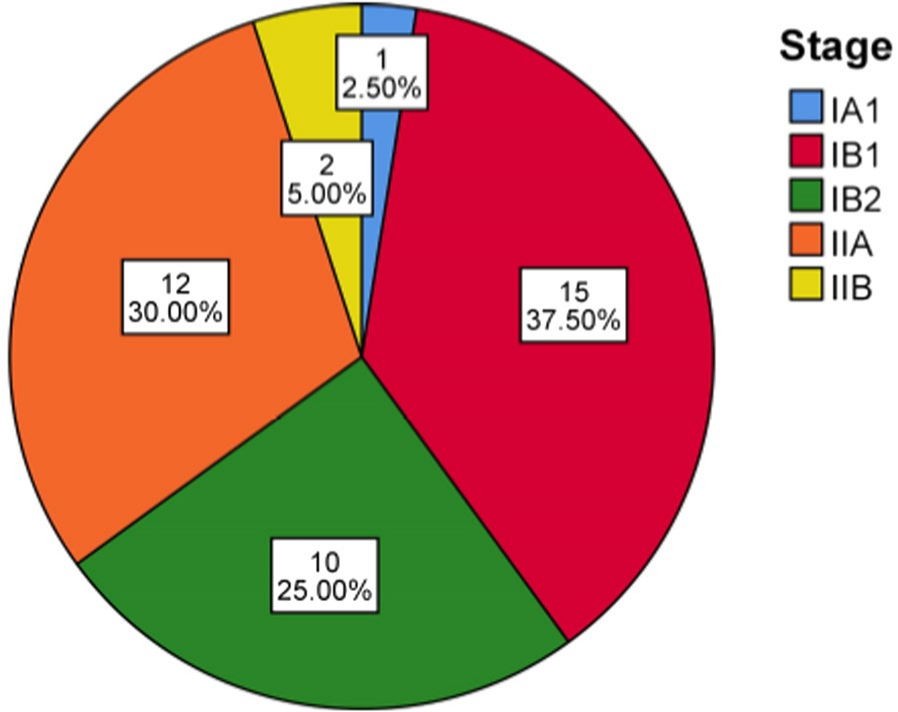
Distribution of cervical cancer stages.

The study comprised 40 patients diagnosed with cervical cancer during pregnancy (38) or the postpartum period (2), from Jan 2010 through June 2019. The clinical stages were IA1 (1/40, 2.5%); IB1 (15/40, 37.5%); IB2 (10/40, 25%); IIA (12/40, 30%); and IIB (2/40, 5%) (Fig. 1). Of the 38 patients diagnosed during pregnancy, the average GA at initial diagnoses was 17.1 weeks; 17 patients were in the first trimester when initially diagnosed, 13 in the second trimester, and 8 in the third trimester. Of the 38 patients, 10 continued the pregnancy (continuation group), including 7 (70%) diagnosed in the third trimester, 3 (30%) in the second trimester. Also, 2 patients diagnosed postpartum are included in this group. The other 28 women terminated their pregnancy after learning of their diagnosis (termination group). The demographic characteristics and clinical information of the 2 groups were compared (Table 1). The average GA at diagnoses of the continuation group and the termination group were 30.3 and 8.6 weeks, respectively. The continuation group was significantly more likely to be diagnosed in the third trimester (V1 vs V3, *P* < 0.01; V2 vs V3, *P* < 0.01). There is no statistical difference between the two groups in average age, age for first sex activity, body mass index (BMI), living status, clinical stage, pathological type, HPV infection, pelvic lymph node metastasis, depth of cervical interstitial invasion, lymphatic vascular space invasion (LVSI), or positive surgical para-uterine infiltration.

**Table 1:** The sociodemographic characteristics and oncological information of the two groups

	Continuation group (N = 12)	Termination group (N = 28)	*P*-value
**Average age**			
Average age(years) M ± SD	31. 58 ± 4. 852	33. 82 ± 4. 982	0. 63
25 ≤ age < 35	9	13	
Age ≥ 35	3	15	0.17
**Age for first sex activity**			
Average age(years) M ± SD	21.00 ± 3.30	20.71 ± 3.219	0.80
15 ≤ age < 20	4	12	
20 ≤ age < 25	6	14	0.69
Age ≥ 25	2	2	
**BMI**	24. 67 ± 2. 19	22. 22 ± 2. 98	0. 07
LS*			
Countryside	5	13	1.00
Town	7	15	
**Clinical stage**			
IA1	0	1	
IB1	6	9	0.75
IB2	2	8	
IIA	4	8	
IIB	0	2	
**Pathological type**			
Squamous cell carcinoma	11	25	
Adenocarcinoma	0	1	0.78
Adenosquamous carcinoma	1	1	
Others	0	1	
**HPV infection**			
HPV 16	6	19	
HPV 18	0	1	0.59
Compound infection	1	1	
Unknown	5	7	
Pelvic lymph*	2	5	1. 00
**LVSI**	6	14	1. 00
**Positive surgical segment**	0	1	1. 00
**Para-uterine infiltration**	1	2	1. 00
Depth of CII*			
≤ 1/2	4	9	1.00
> 1/2	7	17	
**GA at diagnosis**			
V1^*^	0	17	
V2^*^	3	10	< 0.01^①^
V3^*^	7	1	
V4^*^	2	0	
The interval*(days) M ± SD	52.67 ± 40.34	16.26 ± 12.28	< 0.01^②^
EBL*(mL) M ± SD	688. 89 ± 310.02	724. 07 ± 539.12	0. 77^②^
OT* (mins) M ± SD	238. 67 ± 49.34	236. 33 ± 67.52	0. 93
**Follow-up time**(months) M ± SD	61.58 ± 38.58	58.61 ± 35.56	0.57^②^
**Still living** n (%)	11 (100.00)	20 (90.91)	0.54

LS* = living status. The interval* = the interval from the diagnosis to surgery (d); V1* = First trimester. V2* = Second trimester. V3* = Third trimester. V4* = Postpartum. Pelvic lymph* = pelvic lymph nodes metastasis. CII* = depth of cervical interstitial invasion. EBL* = the estimated blood loss. OT* = operation time. ① X^2^ test, than pairwise comparison between groups, ② Rank sum test

### Management during pregnancy

**Fig. 2:**
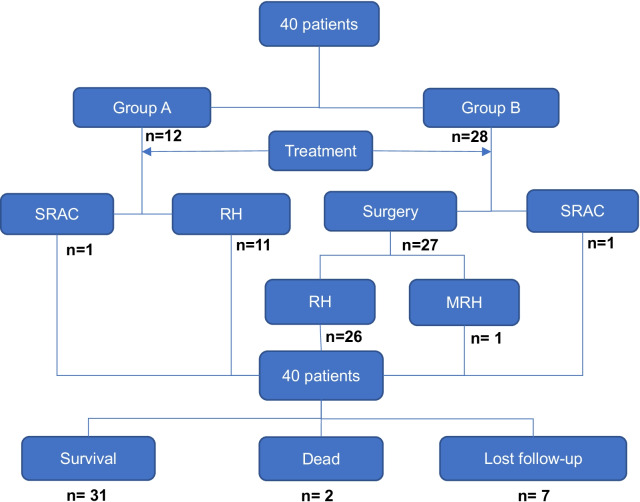
The treatment and prognosis of the 40 patients. MRH = modified radical hysterectomy, RH = radical hysterectomy, SRAC = simultaneous radiotherapy and chemotherapy.

All patients received standard treatment (Fig. 2). 1 patient in each of the 2 groups received simultaneous radiotherapy and chemotherapy without operation. The remaining patients were treated surgically. Except for a single, IA1 patient in the continuation group who underwent modified hysterectomy without pelvic lymph node dissection, the other 37 patients underwent radical hysterectomy including lymph node dissection. 4 patients in the continuation group were treated with NACT during pregnancy to extend gestation and improve fetal maturity. The chemotherapy regimen combined paclitaxel with cisplatin. 1 patient diagnosed at 22 weeks 3 days GA choose to continue pregnancy but refused NACT. The average gestational period at the time of delivery was 36.2 weeks. The mode of delivery of 12 patients was cesarean section (CS) in 11 and vaginal delivery in 1. In our study, 3 patients were diagnosed in the third trimester and treated postpartum. 7 patients were diagnosed in the second or third trimester. Of the 7 patients, 4 received NACT to prolong the gestational age of pregnancy, and 3 received treatment until delivery. All 7 patients underwent surgery at the time of delivery. We found that none of these 10 patients had tumor progression at the time of treatment. 10 patients underwent CS and radical hysterectomy at the same time. 4 patients underwent surgery following delivery and 1 patient received simultaneous radiotherapy and chemotherapy.

### Patient outcomes

The estimated surgical blood loss and operative duration of those 2 groups were similar (postpartum patients excluded). The time from diagnosis to operation in the continuation group was significantly longer than in the termination group (52.7 vs 16.3 days, *P* < 0.01). In the continuation group, 11 patients survived (100%) and 1 was lost to follow-up. In the termination group, 20 patients survived (90.9%), 2 patients died of tumor recurrence, and 6 patients were lost to follow-up. There was no significant difference in survival outcomes between the two groups (*P* = 0.54). All 10 cases that continued the pregnancy had no significant obstetric complications, including cervical insufficiency, preterm labor, preterm premature rupture of membranes, or fetal growth restriction. Pertaining to both groups, there were no peripheral organ injuries (ureteral injury, intestinal fistula, urinary fistula, vascular rupture).

### Fetal outcomes

Of the 2 patients diagnosed postpartum, we unable to determine the gestational age at delivery. Of the 10 cases diagnosed during pregnancy, 6 newborns were premature, and 4 were full-term. The Apgar scores of newborns (1–5–10 min) are shown in Table 2. The Apgar score of all newborns at 10 min after birth was 10. Through neonatal follow-up, 1 newborn was lost follow-up, 1 was diagnosed with lymph node tuberculosis at age 9 and improved with treatment at our hospital. All remaining newborns did not develop tumor, and there were no medical or surgical complications related to NACT.

**Table 2:** The treatment and prognosis of the continuing pregnancy group

No	Stage	Pathological type	GA at diagnosis (w*)	GA at treatment (w*)	GA at surgery (w*)	GA at delivery (w*)	NACT/cycles	POT*	Mode of delivery	The mode of the surgery	The Apgar Score (1–5–10 min)	The follow-up time (month)	The survival condition of the newborns	The outcome of the pregnancy woman
1	IIA	SCC	31 + 3	33 + 2	33 + 2	33 + 2	–	Unknown	CS	RH	8–9–9	117	Lost	Lost
2	IB1	SCC	37 + 0	37 + 6	37 + 6	37 + 6	–	CT + RT	CS	RH	10–10–10	114	Good	Good
3	IB1	SCC	Postpartum 3 months	After diagnose for 6 days	After diagnose for 6 days	–	–	–	CS	RH	10–10–10	101	Good	Good
4	IIA	SCC	Postpartum 3 months	Postpartum 3 months	Postpartum 4 months	–	BVP/2	CT + RT	VD	RH	10–10–10	98	Good	Good
5	IB1	SCC	30 + 6	30 + 6	34 + 3	34 + 3	TP/1	RT	CS	RH	10–10–10	75	Good (Lymphatic tuberculosis)	Good
6	IB1	ASC	38 + 4	Postpartum 12 days	Postpartum 36 days	40 + 0	TP/1	CT	CS	RH	10–10–10	43	Good	Good
7	IB1	SCC	20 + 4	21 + 3	35 + 4	35 + 4	TP/3	RT	CS	RH	10–10–10	39	Good	Good
8	IB2	SCC	32 + 6	33 + 0	36 + 5	36 + 5	TP/1	CT + RT	CS	RH	10–10–10	33	Good	Good
9	IIA	SCC	30 + 1	Postpartum 9 days	No surgery	33 + 1	–	CT + RT	CS	–	10–10–10	31	Good	Good
10	IB1	SCC	22 + 3	34 + 4	34 + 4	34 + 4	Refuse	CT + RT	CS	RH	10–10–10	26	Good	Good
11	IB2	SCC	39 + 4	Postpartum 10 days	Postpartum 48 days	40 + 0	TP/2	CT + RT	CS	RH	10–10–10	15	Good	Good
12	IIA	SCC	20 + 0	25 + 1	26 + 4	36 + 4	TP/3	CT + RT	CS	RH	9–10–10	8	Good	Good

ASC = Adenosquamous carcinoma, BVP = bleomycin + vincristine + cisplatin, CT = Chemotherapy, CT + RT = Chemotherapy and radiotherapy, POT* = Postoperative treatment. w* refers to weeks

## Discussion

Cervical cancer during pregnancy is a rare event. The guidelines for the management of cervical cancer during pregnancy are based on limited data from a small number of cases and expert opinion. Hence, the management of pregnancy complicated by a concomitant diagnosis of cervical cancer remains complex and challenging. Due to delays in childbearing to the third or fourth decade of life, the diagnosis of cervical cancer during pregnancy has risen over recent decades. In addition, the optimal treatment for cervical cancer diagnosed during pregnancy has not been fully clarified. Therefore, studies investigating continuation of pregnancy in patients diagnosed with cervical cancer, specifically detailing outcomes of both mother and fetus, are necessary. In this study, patients who continued the pregnancy showed similar survival compared with patients who terminated the pregnancy. Similarly, the patients who opted to continue the pregnancy showed few obstetric complications, the most common of which was iatrogenic preterm birth that was electively carried out to facilitate treatment. Newborns did not develop medical or surgical complications following NACT. However, cervical cancer during pregnancy remains a difficult problem requiring multidisciplinary discussion. Whether, when and how to terminate a pregnancy, and the impact of treatment on maternal and infant outcomes require a multidisciplinary discussion among obstetrical, gynecological, oncological, and neonatal physicians before further decisions can be made.

Whether continuing pregnancy accelerates the malignancy in the mother is controversial. A review of 76 patients diagnosed with stage IB1 or higher cervical cancer, reported that the survival rate of the patients, with an average delay in treatment of 16 weeks, was 95% [18]. This was taken to indicate that continuation of the pregnancy did not adversely affect maternal oncologic treatment. In contrast, other studies have reported that delayed treatment did aggravate tumor progression, with higher mortality rates and disease recurrences [19, 20]. However, in these studies, the clinical features (such as clinical stage, tumor size, lymph node metastasis) were not compared between the continuation of pregnancy group and the termination of pregnancy group. Furthermore, most of the patients in the studies were stage III and IV, potentially leading to biased results. The staging of cervical cancer should be considered during the treatment of cervical cancer in pregnant women. The 2014 and 2019 International Gynecologic Cancer Society (IGCS) and European Society of Gynecological Oncology (ESGO) guidelines [3, 21], propose less radical surgery (deep cone and simple trachelectomy) for early cervical cancer whose tumor size is smaller than 2 cm. Further studies have confirmed the safety of less radical surgery in non-pregnant women [22, 23]. However, reports have concluded that 1 of 7 (14.3%) cases treated with vaginal radical trachelectomy during pregnancy have resulted in early abortions [24, 25]. Ideally, future prospective studies should be carried to validate these findings. For patients diagnosed after 22 weeks gestation, either NACT or postpartum treatment may be an option. For stage IIA tumors and above, NACT is the only approach to continue the pregnancy.

NACT is an innovative method for the treatment of cervical cancer in pregnant women. This method prevents cervical cancer progression and facilitates delay to delivery in patients whose fetus are not yet mature. The recommended type of NACT for pregnant patients is platinum-based chemotherapy [26]. Teratogenicity of any drug depends on exposure time, the dose, and factors that affect placental transfer. High lipid solubility, low molecular weight, and loose binding to plasma proteins promote transfer of drugs from mother to fetus [27]. Previous studies have reported that the type of fetal deformities is related to the gestational age of exposure to chemotherapeutic drugs. The use of chemotherapy in the first trimester of pregnancy increases the risk of spontaneous abortion, fetal death and severe malformations [27, 28]. Recent studies have shown that the concentrations of chemotherapeutic drugs in the amniotic fluid and umbilical cord blood are significantly lower than in maternal blood when chemotherapy is carried out in the second and third trimesters of pregnancy [10, 29]. In these studies, all 30 of the newborns were born alive without evidence of disease and all children developed normally. However, a systematic review [30] reported that one of 14 neonates whose mother was diagnosed with cervical cancer and treated with NACT was diagnosed with embryonal rhabdomyosarcoma 60 months after delivery, probably due to paclitaxel. Further, another baby developed severe bilateral hearing loss in 6 months after delivery due to cisplatin administration. Overall, the incidence of complications of NACT are low and NACT appears to be a relatively safe method for cervical cancer patients to allow continuation of the pregnancy.

This study has limitations. We carried out a retrospective study which may be affected by confounding and reporting bias. In addition, the sample size was small due to the rarity of cervical cancer occurring during pregnancy. Further, because the study was carried out several years after cervical cancer diagnosis, we were unable to achieve follow-up of all participants.

## Conclusion

The results of this study support the safety of continuation of pregnancy in patients concomitantly diagnosed with cervical cancer. Neither the oncologic outcome of the mother nor surgical or obstetric outcomes compromised using this approach. Additionally, the use of NACT did not threaten the health of the fetus. Therefore, it seems reasonable to continue the pregnancy in patients concomitantly diagnosed with cervical cancer. Clearly, the patient’s individual clinical characteristics must be accounted for and personal preferences must be respected. The findings of our study may be useful in counseling women facing the treatment of cervical cancer diagnosed during pregnancy.

## Data Availability

Research data have been included in this manuscript and supplementary information files.

## Abbreviations

GA: Gestational age
NACT: Neoadjuvant chemotherapy
HPV: Human papillomavirus
CS: Cesarean section
